# Characteristics of The Cancer Genome Atlas cases relative to U.S. general population cancer cases

**DOI:** 10.1038/s41416-018-0140-8

**Published:** 2018-08-21

**Authors:** Xiaoyan Wang, Joseph T. Steensma, Matthew H. Bailey, Qianxi Feng, Hannah Padda, Kimberly J. Johnson

**Affiliations:** 1 Brown School, Washington University in St. Louis, St. Louis MO, USA; 2 Division of Oncology, Department of Medicine, Washington University in St. Louis, St. Louis MO, USA; 3 McDonnell Genome Institute, Washington University in St. Louis, St. Louis MO, USA; 4 Siteman Cancer Center, Washington University in St. Louis, St. Louis MO, USA

**Keywords:** Cancer epidemiology, Cancer genomics

## Abstract

**Background:**

Despite anecdotal reports of differences in clinical and demographic characteristics of The Cancer Genome Atlas (TCGA) relative to general population cancer cases, differences have not been systematically evaluated.

**Methods:**

Data from 11,160 cases with 33 cancer types were ascertained from TCGA data portal. Corresponding data from the Surveillance, Epidemiology, and End Results (SEER) 18 and North American Association of Central Cancer Registries databases were obtained. Differences in characteristics were compared using Student’s *t*, Chi-square, and Fisher’s exact tests. Differences in mean survival months were assessed using restricted mean survival time analysis and generalised linear model.

**Results:**

TCGA cases were 3.9 years (95% CI 1.7–6.2) younger on average than SEER cases, with a significantly younger mean age for 20/33 cancer types. Although most cancer types had a similar sex distribution, race and stage at diagnosis distributions were disproportional for 13/18 and 25/26 assessed cancer types, respectively. Using 12 months as an end point, the observed mean survival months were longer for 27 of 33 TCGA cancer types.

**Conclusions:**

Differences exist in the characteristics of TCGA vs. general population cancer cases. Our study highlights population subgroups where increased sample collection is warranted to increase the applicability of cancer genomic research results to all individuals.

## Introduction

In recent years, progress in genome sequencing technologies and bioinformatics has provided enormous gains in understanding of the molecular aberrations associated with the development of various cancers. The emergence of publicly available cancer genomic datasets, including The Cancer Genome Atlas (TCGA), facilitates the comprehensive understanding of the molecular pathogenesis of cancer and is allowing for the development of new strategies to improve cancer diagnosis, therapy, and prevention. By analysing these publicly available genomic data, many novel disease-associated genes have been uncovered.^1,2^

TCGA was formed in 2005 when the U.S. National Cancer and National Human Genome Research Institutes teamed together to support the launch of the project to comprehensively map various cancer genomic changes. To date, more than 11,000 individuals  with 33 cancer types have been included in the cohort.^3,4^ These data have thus far contributed to >2000 studies of various cancers in PubMed.

The cohort composition for each cancer type is an important consideration since the results generated from these cases may be used to make inferences about the respective cancer type among the general population. Prior studies have shown that race, which is often used as a proxy for ancestry and social exposures, is related to the pathogenesis of cancer and different genetic backgrounds in common tumour types may influence clinical outcome and response to therapy.^5–7^ Evidence has shown that somatic mutation frequency differs by race in various cancer types,^8–10^ implying that factors associated with race can impact the somatic mutation landscape. Other evidence also highlights the implications of sex and age dissimilarities in genetic susceptibility to cancer.^11–13^ For these reasons and because TCGA data was assembled mainly from an eligible convenience sample of cancer patients with strict sample selection criteria,^14^ it is important to understand similarities and differences in the characteristics of individuals who have contributed samples to TCGA relative to those of the general population of individuals diagnosed with cancer. A previous study of TCGA cases found that race/ethnicity disparities exist relative to the U.S. general population for ten cancer types examined comprising 5729 cases.^15^ Another study that analysed nine different cancer types in TCGA indicated a dissimilar age distribution in comparison with corresponding cases in the Surveillance, Epidemiology, and End Results (SEER) database.^16^ However, differences in demographic and clinical characteristics beyond race/ethnicity and age between members of the TCGA and the general U.S. population of cancer cases have not been systematically characterised.

In this study, we extend the results from previous studies by comparing demographic and clinical characteristics (age at diagnosis, sex, race, stage at diagnosis, and survival months) between TCGA cases with 33 cancer types and cases in two population-based databases: (1) the SEER 18 database that currently covers ~28% of the U.S. population,^17^ and (2) the U.S. combined registries of North American Association of Central Cancer Registries (NAACCR) that covers cancer registrations in all 50 states and the District of Columbia.^18^

## Methods

### Population

Three separate data sources were used in this study: TCGA,^19^ the SEER 18 database,^17^ and the NAACCR public use dataset.^20^ Data from individuals diagnosed with 33 cancer types were extracted from TCGA. No duplicate cases were found across various cancer types as determined by matching TCGA case IDs. Individuals with corresponding cancer types in SEER were identified using the third edition of the International Classification of Diseases for Oncology (ICD-O-3) by primary site and histology/behavior (Supplementary Table 1). To compare TCGA cases to a contemporary population of individuals diagnosed with cancer, only cases diagnosed with a primary malignancy from 2010 to 2013 in SEER were included. Since SEER intentionally oversamples U.S. minority populations,^21^ we used data from NAACCR to compare race distributions. This public use dataset published in the annual Cancer in North American (CiNA) Volumes covers cancer registrations in all 50 states and the District of Columbia, approaching 100% coverage of the U.S. population in the most recent time period.^22^ The most current five  years (2009–2013) of data for U.S. and Canadian individuals diagnosed with cancer  were available in this dataset. In this study, only U.S. cancer cases  with available race data were included. The corresponding cancer types in NAACCR were defined using the cancer sites as denoted in Supplementary Table 1.

### Variables

XML files from TCGA containing data on demographics, cancer variables, and follow-up status were downloaded from the National Cancer Institute Genomic Data Commons data portal^19^ on 22 December 2016. Python 3.6.0 was used to parse these files and extract the variables. Demographic data including diagnosis age, sex, and race were extracted from the “clin_shared:age_at_initial_pathologic_diagnosis”, “shared:gender”, “clin_shared:race” fields. Race was categorised as White, Black (African American), and Other (Asian, American Indian, or Alaska Native). Ethnicity was not included in this analysis due to the large proportion (24%) of cases with missing data for this field. Clinical information was extracted from the “shared_stage:clinical_stage”, “shared_stage:pathological_stage”, “shared_last_contact_days_to”, and “shared_death_days_to” fields. Stage was defined according to American Joint Committee on Cancer (AJCC) staging that includes categories I, II, III, and IV. Survival months were calculated using the “shared_last_contact_days_to” field for cases who were still alive and “shared_death_days_to field” for cases who were deceased during the follow-up period divided by days in a month (365.24/12).^23^ Similarly, the demographic and clinical data of the 33 corresponding cancers were extracted from the SEER 18 database using SEER*Stat 8.3.4. Diagnosis age was based on the SEER variable “Age at diagnosis”. Race classifications were based on the “Race recode (W, B, AI, API)” variable and defined the same as above. Stage at diagnosis was defined using the “Derived AJCC Stage Group (7th edition 2010+)” variable. Survival months were defined using the “Survival months” variable. In NAACCR, the race categories were based on the “Race (Includes Hispanic)” variable and defined the same as for TCGA.

### Statistical analysis

Stata version 14 was used for all analyses. Student’s *t*-test and Cohen’s d, a measure of effect size, were used to identify and quantify the statistical differences and effect sizes in diagnosis age. Cohen’s d is calculated as the difference between two means divided by the pooled standard deviation.^24^ By convention, Cohen’s d ≥ 0.3 indicates at least a moderate effect size. Ordinary least squares regression was used to estimate the overall mean difference in diagnosis age between TCGA and SEER cases with adjustment for cancer types. Chi-square and Fisher’s exact tests were used to identify proportion differences in sex, race, and stage. Additionally, for race and stage comparisons, adjusted residuals were used to determine categories with the largest difference relative to sample size. An adjusted residual ≥ 2.0 indicates that there was a significantly greater proportion of a particular race or stage category among TCGA cases than in the comparison population (i.e., NAACCR or SEER), while an adjusted residual ≤ −2.0 indicates a significantly lower proportion. We also quantified the mean all-cause survival months using restricted mean survival time (RMST) analysis^25^ using 12 months as the end point to ensure that all TCGA cases that were included have the same window of observation. Since all TCGA cases were diagnosed prior to 2014, all had at least 12 months of follow-up time except for the cases who died during this period. For cases with over 12 survival months, the survival months were truncated at 12. The RMST approach is valid for any distribution of time to event.^25–29^ The between-group difference in mean survival with corresponding 95% confidence intervals (CIs) was estimated at 12-month horizon with adjustment for diagnosis age, sex, race, and stage if available for a specific cancer type, and a subsequent generalised linear model with robust standard errors. Statistical tests for all analyses were two-tailed tests and the critical value for alpha for all tests was 0.05.

## Results

Of 11,160 TCGA cases with 33 cancer types diagnosed between 1978 and 2013, 1097 cases were diagnosed with breast invasive carcinoma (BRCA) followed by glioblastoma multiforme (GBM, *n* = 596), ovarian serous cystadenocarcinoma (OV, *n* = 587), uterine corpus endometrial carcinoma (UCEC, *n* = 548), kidney renal clear cell carcinoma (KIRC, *n* = 537), head and neck squamous cell carcinoma (HNSC, *n* = 528), lung adenocarcinoma (LUAD, *n* = 522), and brain lower grade glioma (LGG, *n* = 515). Six cancers including adrenocortical carcinoma (ACC), cholangiocarcinoma (CHOL), lymphoid neoplasm diffuse large B-cell lymphoma (DLBC), mesothelioma (MESO), uterine carcinosarcoma (UCS), and uveal melanoma (UVM) had < 100 cases each. Among the corresponding 33 diagnoses in SEER, the number of cases ranged from 164 (UCS) to 203,828 (BRCA). In NAACCR, the number of cases ranged from 15,705 (MESO) to 1,085,443 (BRCA). Demographic and clinical characteristics of TCGA and SEER cases are shown in Table 1. The race distribution of TCGA and NAACCR cases is shown in Table 2.

**Table 1:** Demographic and clinical characteristics of TCGA and SEER cases

Cancer types	Age		Male		Stage								Restricted mean survival months	
					I		II		III		IV			
	TCGA^a^ *N* (mean, SD)	SEER *N* (mean, SD)	TCGA *N* (%)	SEER *N* (%)	TCGA *N* (%)	SEER *N* (%)	TCGA *N* (%)	SEER *N* (%)	TCGA *N* (%)	SEER *N* (%)	TCGA *N* (%)	SEER *N* (%)	TCGA *N* (mean, SD)	SEER *N* (mean, SD)
All cancers	11,109 (59.1, 14.4)	1,027,049 (63.0, 13.9)	—	—	—	—	—	—	—	—	—	—	—	—
ACC	92 (47.2, 16.3)	349 (52.5, 20.0)	32 (34.8)	131 (37.5)	9 (10.0)	15 (4.4)	44 (48.9)	103 (30.5)	19 (21.1)	48 (14.2)	18 (20.0)	172 (50.9)	79 (11.7, 1.3)	337 (8.9, 4.3)
BLCA	412 (68.1, 10.6)	49,593 (70.1, 12.1)	304 (73.8)	37,796 (76.2)	2 (0.5)	10,796 (51.9)	131 (32.0)	5353 (25.7)	141 (34.4)	1644 (7.9)	136 (33.2)	3005 (14.5)	391 (11.1, 2.3)	20,621 (10.4, 3.3)
BRCA	1096 (58.4, 13.2)	203,828 (60.7, 13.6)	12 (1.1)	1474 (0.7)	183 (17.1)	97,507 (49.5)	621 (57.9)	65,060 (33.0)	249 (23.1)	23,245 (11.8)	20 (1.9)	11,363 (5.8)	979 (11.9, 0.5)	195,859 (11.8, 1.5)
CESC	307 (48.3, 13.8)	11,454 (50.1, 14.7)	—	—	163 (54.3)	5343 (49.0)	70 (23.3)	1494 (13.7)	46 (15.3)	2471 (22.6)	21 (7.0)	1606 (14.7)	259 (11.6, 1.4)	10,799 (11.2, 2.5)
CHOL	45 (63.6, 12.2)	4743 (67.3, 13.2)	20 (44.4)	2363 (49.8)	20 (44.4)	569 (17.6)	11 (24.4)	541 (16.8)	4 (8.9)	270 (8.4)	10 (22.2)	1846 (57.2)	43 (10.6, 3.3)	3214 (7.0, 4.7)
COAD	459 (66.9, 13.1)	78,858 (66.7, 14.1)	243 (52.9)	38,938 (49.4)	76 (17.0)	17,127 (23.2)	178 (39.7)	20,294 (27.5)	129 (28.8)	19,987 (27.1)	65 (14.5)	16,464 (22.3)	268 (11.6, 1.7)	73,335 (10.5, 3.5)
DLBC	48 (55.3, 14.2)	20,129 (63.7, 16.7)	22 (45.8)	11,040 (54.9)	8 (19.1)	2937 (17.3)	17 (40.5)	3916 (23.1)	5 (11.9)	3497 (20.6)	12 (28.6)	6604 (39.0)	41 (11.9, 0.4)	16,804 (9.7, 4.1)
ESCA	185 (62.5, 11.9)	12,068 (66.3, 11.7)	158 (85.4)	9617 (79.7)	18 (11.1)	1934 (18.3)	79 (48.8)	1860 (17.6)	56 (34.6)	2927 (27.7)	9 (5.6)	3854 (36.4)	143 (10.8, 2.6)	10,429 (8.2, 4.4)
GBM	596 (57.8, 14.4)	9818 (62.1, 14.7)	366 (61.4)	5628 (57.3)	—	—	—	—	—	—	—	—	566 (9.1, 3.9)	9743 (7.7, 4.5)
HNSC	527 (60.9, 11.9)	36,907 (62.5, 12.2)	386 (73.1)	27,933 (75.7)	27 (6.0)	7330 (22.4)	74 (16.3)	3924 (12.0)	82 (18.1)	5589 (17.1)	270 (59.6)	15,873 (48.5)	437 (11.1, 2.4)	32,482 (10.8, 2.9)
KICH	113 (51.2, 13.9)	2151 (58.6, 13.9)	62 (54.9)	1185 (55.1)	54 (47.8)	1380 (65.6)	33 (29.2)	377 (17.9)	19 (16.8)	303 (14.4)	7 (6.2)	45 (2.1)	110 (11.9, 1.1)	2076 (11.9, 0.9)
KIRC	537 (60.6, 12.2)	22,371 (60.3, 12.4)	346 (64.4)	13,855 (61.9)	269 (50.4)	13,769 (62.7)	57 (10.7)	1913 (8.7)	125 (23.4)	3722 (17.0)	83 (15.5)	2550 (11.6)	522 (11.4, 2.1)	21,768 (11.5, 2.1)
KIRP	288 (61.5, 12.1)	4576 (61.3, 11.9)	214 (73.5)	3406 (74.4)	173 (66.3)	3249 (73.7)	21 (8.1)	449 (10.2)	52 (19.9)	447 (10.1)	15 (5.8)	266 (6.0)	244 (11.8, 0.9)	4362 (11.5, 2.1)
LAML	200 (55.0, 16.1)	11,671 (60.3, 21.7)	109 (54.5)	6336 (54.3)	—	—	—	—	—	—	—	—	173 (9.6, 3.6)	11,491 (7.0, 5.0)
LGG	515 (42.9, 13.4)	952 (35.4, 20.6)	285 (55.3)	536 (56.3)	—	—	—	—	—	—	—	—	500 (11.7, 1.4)	943 (11.4, 2.3)
LIHC	376 (59.5, 13.5)	22,642 (63.1, 11.0)	255 (67.6)	17,529 (77.4)	175 (49.6)	7604 (40.3)	87 (24.7)	3774 (20.0)	86 (24.4)	3520 (18.7)	5 (1.4)	3953 (21.0)	320 (11.1, 2.7)	18,716 (7.9, 4.8)
LUAD	503 (65.3, 10.0)	69,483 (67.4, 11.5)	242 (46.4)	33,003 (47.5)	279 (54.3)	15,333 (22.8)	124 (24.1)	5027 (7.5)	85 (16.5)	10,599 (15.8)	26 (5.1)	36,182 (53.9)	444 (11.3, 1.9)	66,875 (8.3, 4.6)
LUSC	495 (67.3, 8.6)	31,355 (69.7, 10.1)	373 (74.0)	19,553 (62.4)	245 (49.0)	6069 (20.3)	163 (32.6)	3803 (12.7)	85 (17.0)	8815 (29.5)	7 (1.4)	11173 (37.4)	381 (10.8, 2.7)	29,771 (7.9, 4.6)
MESO	87 (63.0, 9.8)	2561 (71.4, 12.3)	71 (81.6)	1928 (75.3)	10 (11.5)	437 (21.7)	16 (18.4)	221 (11.0)	45 (51.7)	466 (23.1)	16 (18.4)	892 (44.3)	85 (10.2, 3.1)	2011 (7.5, 4.5)
OV	587 (59.7, 11.5)	5126 (62.7, 12.6)	—	—	6 (1.1)	456 (9.1)	30 (5.3)	400 (8.0)	446 (78.1)	2669 (53.2)	89 (15.6)	1495 (29.8)	543 (11.3, 2.5)	5003 (10.9, 3.0)
PAAD	185 (64.9, 11.1)	29,367 (67.2, 12.1)	102 (55.1)	15,141 (51.7)	21 (11.5)	2587 (9.3)	152 (83.5)	8036 (28.7)	4 (2.2)	2708 (9.7)	5 (2.8)	14,629 (52.3)	173 (10.3, 2.9)	27,851 (6.8, 4.7)
PCPG	179 (47.3, 15.1)	176 (50.2, 18.9)	78 (43.6)	91 (51.7)	—	—	—	—	—	—	—	—	175 (11.9, 1.0)	166 (11.3, 2.5)
PRAD	500 (61.0, 6.8)	195,005 (65.7, 9.1)	—	—	—	—	—	—	—	—	—	—	156 (12.0, 0.0)	186,537 (11.8, 1.1)
READ	170 (64.5, 11.9)	26,251 (61.5, 13.5)	92 (54.1)	15,357 (58.5)	33 (20.5)	7346 (32.7)	51 (31.7)	4705 (21.0)	52 (32.3)	6335 (28.2)	25 (15.5)	4065 (18.1)	80 (11.9, 0.2)	22,242 (11.1, 2.6)
SARC	261 (60.9, 14.7)	3256 (59.3, 20.4)	119 (45.6)	1734 (53.3)	—	—	—	—	—	—	—	—	252 (11.6, 1.7)	3158 (9.2, 4.4)
SKCM	462 (58.2, 15.7)	61,799 (59.9, 16.3)	290 (61.7)	34,964 (56.6)	91 (21.4)	43,082 (76.2)	140 (32.9)	6950 (12.3)	171 (40.2)	4321 (7.6)	23 (5.4)	2205 (3.9)	405 (11.8, 0.9)	53,395 (11.7, 1.7)
STAD	438 (65.7, 10.8)	18,809 (66.2, 14.3)	285 (64.3)	11,540 (61.4)	59 (14.2)	3631 (22.2)	130 (31.3)	2552 (15.6)	183 (44.0)	3624 (22.2)	44 (10.6)	6536 (40.0)	340 (10.8, 2.4)	16,226 (8.4, 4.5)
TGCT	134 (32.0, 9.3)	8966 (34.4, 11.8)	—	—	101 (79.5)	6145 (72.2)	12 (9.5)	1170 (13.7)	14 (11.0)	1201 (14.1)	—	—	122 (11.9, 1.0)	8170 (11.9, 1.1)
THCA	507 (47.3, 15.8)	43,969 (49.1, 15.3)	136 (26.8)	10,040 (22.8)	285 (56.4)	30,698 (72.1)	52 (10.3)	3162 (7.4)	113 (22.4)	5387 (12.7)	55 (10.9)	3333 (7.8)	412 (12.0, 0.4)	41,825 (11.8, 1.2)
THYM	123 (58.2, 13.0)	584 (58.2, 14.1)	64 (51.6)	268 (45.9)	—	—	—	—	—	—	—	—	121 (11.9, 0.7)	575 (11.5, 1.9)
UCEC	545 (63.9, 11.1)	36,753 (61.5, 11.8)	—	—	342 (62.4)	27,851 (78.8)	52 (9.5)	1657 (4.7)	124 (22.6)	3795 (10.7)	30 (5.5)	2048 (5.8)	514 (11.8, 1.1)	35,073 (11.6, 1.7)
UCS	57 (69.7, 9.3)	164 (65.4, 11.1)	—	—	22 (38.6)	20 (13.3)	5 (8.8)	11 (7.3)	20 (35.1)	47 (31.3)	10 (17.5)	72 (48.0)	55 (10.8, 2.8)	138 (9.1, 4.)
UVM	80 (61.7, 13.9)	1315 (60.2, 14.7)	45 (56.3)	681 (51.8)	0	308 (33.2)	39 (49.4)	463 (50.0)	36 (45.6)	134 (14.5)	4 (5.1)	22 (2.4)	54 (11.6, 1.7)	911 (11.8, 1.1)

*ACC* adrenocortical carcinoma, *BLCA* bladder urothelial carcinoma, *BRCA* breast invasive carcinoma, *CESC* cervical squamous cell carcinoma and endocervical adenocarcinoma, *CHOL* cholangiocarcinoma, *COAD* colon adenocarcinoma, *DLBC* lymphoid neoplasm diffuse large B-cell lymphoma, *ESCA* oesophageal carcinoma, *GBM* glioblastoma multiforme, *HNSC* head and neck squamous cell carcinoma, *KICH* kidney chromophobe, *KIRC* kidney renal clear cell carcinoma, *KIRP* kidney renal papillary cell carcinoma, *LAML* acute myeloid leukaemia, *LGG* brain lower grade glioma, *LIHC* liver hepatocellular carcinoma, *LUAD* lung adenocarcinoma, *LUSC* lung squamous cell carcinoma, *MESO* mesothelioma, *OV* ovarian serous cystadenocarcinoma, *PAAD* pancreatic adenocarcinoma, *PCPG* pheochromocytoma and paraganglioma, *PRAD* prostate adenocarcinoma, *READ* rectum adenocarcinoma, *SARC* sarcoma, *SKCM* skin cutaneous melanoma, *STAD* stomach adenocarcinoma, *TGCT* testicular germ cell tumours, *THCA* thyroid carcinoma, *THYM* thymoma, *UCEC* uterine corpus endometrial carcinoma, *UCS* uterine carcinosarcoma, *UVM* uveal melanoma, *SD* standard deviation.

^a^All cancer types have no missing values for age in TCGA except for BRCA (one case missing), HNSC (one case missing), KIRP (three cases missing), LIHC (one case missing), LUAD (nineteen cases missing), LUSC (nine cases missing), SKCM (eight cases missing), STAD (five cases missing), THYM (one case missing), and UCEC (three cases missing)

**Table 2:** Race distribution of TCGA and NAACCR cases

Cancer types	Total		White		Black		Other	
	TCGA *N*	NAACCR *N*	TCGA *N* (%)	NAACCR *N* (%)	TCGA *N* (%)	NAACCR *N* (%)	TCGA *N* (%)	NAACCR *N* (%)
All cancers	5899	4,834,006	4845 (82.1)	4,118,891 (85.2)	500 (8.5)	548,447 (11.3)	554 (9.4)	166,668 (3.4)
BLCA	394	335,620	327 (83.0)	310,352 (92.5)	23 (5.8)	18,912 (5.6)	44 (11.2)	6356 (1.9)
BRCA	1002	1,085,443	758 (75.7)	913,884 (84.2)	183 (18.3)	128,208 (11.8)	61 (6.1)	43,351 (4.0)
CESC	271	59,092	221 (81.6)	46,030 (77.9)	30 (11.1)	9902 (16.8)	20 (7.4)	3160 (5.4)
COAD	285	477,271	215 (75.4)	397,760 (83.3)	59 (50.6)	62,360 (13.1)	11 (3.9)	17,151 (3.6)
ESCA	165	78,445	114 (69.1)	68,590 (87.4)	5 (3.0)	8034 (10.2)	46 (27.9)	1821 (2.3)
HNSC	513	416,929	454 (88.5)	364,624 (87.5)	48 (9.4)	40,315 (9.7)	11 (2.1)	11,990 (2.9)
LAML	198	65,266	181 (91.4)	56,612 (86.7)	15 (7.6)	5983 (9.2)	2 (1.0)	2671 (4.1)
LIHC	367	131,074	189 (51.5)	100,398 (76.6)	17 (4.6)	19,865 (15.2)	161 (43.9)	10,811 (8.3)
MESO	87	15,705	85 (97.7)	14,745 (93.9)	1 (1.2)	740 (4.7)	1 (1.2)	220 (1.4)
OV	556	102,308	502 (90.3)	88,406 (86.4)	34 (6.1)	9521 (9.3)	20 (3.6)	4381 (4.3)
PAAD	180	205,375	162 (90.0)	172,558 (84.0)	7 (3.9)	25,856 (12.6)	11 (6.1)	6961 (3.4)
PRAD	156	944,300	147 (94.2)	768,438 (81.4)	7 (4.5)	153,082 (16.2)	2 (1.3)	22,780 (2.4)
READ	89	188,774	82 (92.1)	157,903 (83.7)	6 (6.7)	21,534 (11.4)	1 (1.1)	9337 (5.0)
SARC	252	51,566	228 (90.5)	43,208 (83.8)	18 (7.1)	6336 (12.3)	6 (2.4)	2022 (3.9)
SKCM	460	314,232	447 (97.2)	311,095 (99.0)	1 (0.2)	1747 (0.6)	12 (2.6)	1390 (0.4)
STAD	381	108,778	279 (73.2)	83,030 (76.3)	13 (3.4)	17,535 (16.1)	89 (23.4)	8213 (7.6)
TGCT	129	38,765	119 (92.3)	36,311 (93.7)	6 (4.7)	1380 (3.6)	4 (3.1)	1074 (2.8)
THCA	414	215,063	335 (80.9)	184,947 (86.0)	27 (6.5)	17,137 (8.0)	52 (12.6)	12,979 (6.0)

*BLCA* bladder urothelial carcinoma, *BRCA* breast invasive carcinoma, *CESC* cervical squamous cell carcinoma and endocervical adenocarcinoma, *COAD* colon adenocarcinoma, *ESCA* oesophageal carcinoma, *HNSC* head and neck squamous cell carcinoma, *LAML* acute myeloid leukaemia, *LIHC* liver hepatocellular carcinoma, *MESO* mesothelioma, *OV* ovarian serous cystadenocarcinoma, *PAAD* pancreatic adenocarcinoma, *PRAD* prostate adenocarcinoma, *READ* rectum adenocarcinoma, *SARC* sarcoma, *SKCM* skin cutaneous melanoma, *STAD* stomach adenocarcinoma, *TGCT* testicular germ cell tumours, *THCA* thyroid carcinoma.

*Note*: 18 cancer types were included

### Age at diagnosis

**Fig. 1:**
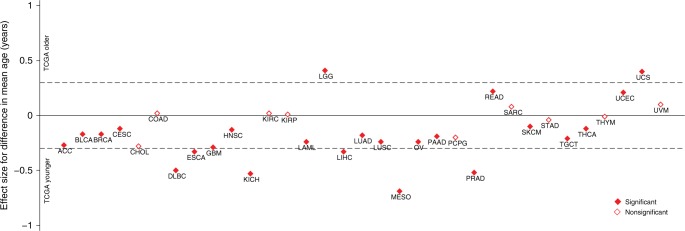
Age at diagnosis difference between TCGA and SEER cases. Filled diamonds indicate a statistically significant difference (*P* < 0.05). The *y*-axis shows the effect size in terms of Cohen’s d. Cohen’s d was calculated as the difference between the mean diagnosis age for TCGA and SEER cases divided by the pooled standard deviation^24^ of each cancer with Cohen’s d > |±0.3| indicating at least a moderate effect size. Cohen’s d < 0 indicates TCGA cases with a younger mean age than SEER cases

Overall, the mean diagnosis age of TCGA cases was 3.9 years younger (95% CI: 1.7–6.2, *P* *<* 0.001) than SEER cases after adjusting for cancer types (data not shown). The mean diagnosis age of TCGA cancer cases  was not significantly different from that of SEER cases for a minority of cancers (CHOL, colon adenocarcinoma (COAD), KIRC, kidney renal papillary cell carcinoma (KIRP), pheochromocytoma and paraganglioma (PCPG), sarcoma (SARC), stomach adenocarcinoma (STAD), thymoma (THYM), and UVM). In contrast, for most cancer types (24/33), there were statistically significant differences in the mean diagnosis age. Among these, the majority (20/24) had a significantly younger mean diagnosis age with the exceptions of LGG, rectum adenocarcinoma (READ), UCEC, and UCS, TCGA cases that had statistically significant older mean diagnosis age than SEER cases (Fig. 1). The difference in the mean diagnosis age was especially pronounced for DLBC (8.4 ± 2.4 years younger in TCGA), oesophageal carcinoma (ESCA, 3.8 ± 0.9 years younger), kidney chromophobe (KICH, 7.4 ± 1.3 years younger), LGG (7.5 ± 0.9 years older), liver hepatocellular carcinoma (LIHC, 3.6 ± 0.7 years younger), MESO (8.4 ± 1.3 years younger), prostate adenocarcinoma (PRAD, 4.7 ± 0.4 years younger), and UCS (4.3 ± 1.5 years older) cases where the absolute effect size for the diagnosis age difference (Cohen’s d) was ≥ 0.3 (Table 3).

**Table 3:** Differences of demographic and clinical characteristics distribution among TCGA, SEER, and NAACCR cases^a^

Cancer types	Age		Sex	Race				Stage					Survival months
	Cohen’s d	*P* ^b^	*P* ^c^	White^d^	Black^d^	Other^d^	*P* ^c^	I^d^	II^d^	III^d^	IV^d^	*P* ^c^	Difference in mean survival (95% CI)
ACC	−**0.27**	**0.009**	0.63	—	—	—	—	**2.04**	**3.27**	1.6	−**5.24**	**<0.001**	**1.46 (0.87, 2.06)** ^e^
BLCA	−**0.17**	**<0.001**	0.25	−**7.12**	0.17	**13.46**	**<0.001**	−**20.63**	**2.85**	**19.13**	**10.57**	**<0.001**	**1.54 (1.29, 1.80)** ^e^
BRCA	−**0.17**	**<0.001**	0.15	−**7.41**	**6.32**	**3.38**	**<0.001**	−**21.17**	**17.27**	**11.54**	−**5.48**	**<0.001**	**0.11 (0.08, 0.15)** ^e^
CESC	−**0.12**	**0.04**	—	1.5	−**2.45**	1.48	**0.02**	1.84	**4.76**	−**2.99**	−**3.74**	**<0.001**	0.11 (-0.07, 0.30)^f^
CHOL	−0.28	0.07	0.47	—	—	—	—	**4.65**	1.37	0.13	−**4.71**	**<0.001**	**2.35 (1.32, 3.39)** ^e^
COAD	0.02	0.73	0.13	−**3.58**	**3.82**	0.24	**0.001**	−**3.11**	**5.79**	0.83	−**3.95**	**<0.001**	**0.73 (0.51, 0.96)** ^e^
DLBC	−**0.5**	**<0.001**	0.21	—	—	—	—	0.3	**2.67**	−1.4	−1.38	**0.04**	**1.37 (0.95, 1.78)** ^e^
ESCA	−**0.33**	**<0.001**	0.06	−**7.09**	−**3.05**	**21.54**	**<0.001**	−**2.35**	**10.24**	1.94	−**8.13**	**<0.001**	**0.89 (0.44, 1.35)** ^e^
GBM	−**0.29**	**<0.001**	0.05	—	—	—	—	—	—	—	—	—	**0.90 (0.58, 1.22)** ^g^
HNSC	−**0.13**	**0.003**	0.17	0.71	−0.24	−0.99	0.59	−**8.37**	**2.82**	0.57	**4.69**	**<0.001**	**0.42 (0.19, 0.64)** ^e^
KICH	−**0.53**	**<0.001**	0.96	—	—	—	—	−**3.85**	**3.01**	0.71	**2.78**	**<0.001**	0.07 (−0.17, 0.31)^e^
KIRC	0.02	0.57	0.24	—	—	—	—	−**5.82**	1.58	**3.91**	**2.79**	**<0.001**	0.09 (−0.10, 0.27)^e^
KIRP	0.01	0.84	0.74	—	—	—	—	−**2.62**	−1.13	**4.98**	−0.19	**<0.001**	**0.36 (0.24, 0.49)** ^e^
LAML	−**0.24**	**<0.001**	0.95	1.94	−0.77	−2.19	0.06	—	—	—	—	—	**1.86 (1.34, 2.39)** ^g^
LGG	**0.41**	**<0.001**	0.72	—	—	—	—	—	—	—	—	—	**0.52 (0.30, 0.74)** ^g^
LIHC	−**0.33**	**<0.001**	**<0.001**	−**11.33**	−**5.61**	**24.64**	**<0.001**	**3.5**	**2.15**	**2.71**	−**9**	**<0.001**	**1.77 (1.43, 2.11)** ^e^
LUAD	−**0.18**	**<0.001**	0.6	—	—	—	—	**16.86**	**14.17**	0.47	−**22.11**	**<0.001**	**0.48 (0.28, 0.69)** ^e^
LUSC	−**0.24**	**<0.001**	**<0.001**	—	—	—	—	**15.67**	**13.07**	−**6.1**	−**16.56**	**<0.001**	**0.66 (0.37, 0.95)** ^e^
MESO	−**0.69**	**<0.001**	0.18	1.48	−1.57	−0.2	0.29	−**2.27**	**2.15**	**6.09**	−**4.77**	**<0.001**	**1.60 (0.86, 2.34)** ^e^
OV	−**0.24**	**<0.001**	—	**2.67**	−**2.59**	−0.8	**0.02**	−**6.61**	−**2.31**	**11.37**	−**7.13**	**<0.001**	0.09 (−0.14, 0.31)^f^
PAAD	−**0.19**	**0.01**	0.33	**2.19**	−**3.52**	**2.02**	**<0.001**	1.06	**16.22**	−**3.41**	−**13.34**	**<0.001**	**1.13 (0.66, 1.61)** ^e^
PCPG	−0.17	0.12	0.13	—	—	—	—	—	—	—	—	—	**0.54 (0.14, 0.95)** ^g^
PRAD	−**0.52**	**<0.001**	—	**4.12**	−**3.97**	0.92	**<0.001**	—	—	—	—	—	**0.05 (0.03, 0.07)** ^**h**^
READ	**0.22**	**0.002**	0.25	2.17	−1.39	−1.67	0.08	−**3.3**	**3.33**	1.15	−0.85	**0.001**	**0.76 (0.56, 0.95)** ^e^
SARC	0.08	0.12	**0.02**	**2.88**	−**2.48**	−1.26	**0.02**	—	—	—	—	—	**2.47 (2.19, 2.74)** ^g^
SKCM	−**0.1**	**0.03**	**0.03**	−**3.69**	−1.04	**6.68**	**<0.001**	−**26.25**	**12.85**	**24.84**	1.6	**<0.001**	**0.37 (0.24, 0.51)** ^e^
STAD	−0.04	0.31	0.2	−**1.42**	−**6.74**	**11.62**	**<0.001**	−**3.91**	**8.59**	**10.49**	−**12.13**	**<0.001**	**0.89 (0.62, 1.15)** ^e^
TGCT	−**0.21**	**0.003**	—	0.66	0.67	0.23	0.78	1.84	−1.4	−0.99	—	0.18	0.0003 (−0.18, 0.18)^f^
THCA	−**0.12**	**0.008**	**0.03**	−**2.97**	−1.09	**5.57**	**<0.001**	−**7.78**	**2.44**	**6.51**	**2.54**	**<0.001**	**0.15 (0.09, 0.21)** ^e^
THYM	−0.01	0.95	0.25	—	—	—	—	—	—	—	—	—	**0.35 (0.14, 0.57)** ^g^
UCEC	**0.21**	**<0.001**	—	—	—	—	—	−**9.27**	**5.24**	**8.86**	−0.32	**<0.001**	**0.29 (0.18, 0.40)** ^f^
UCS	**0.4**	**0.005**	—	—	—	—	—	**4.04**	0.35	0.52	−**4**	**<0.001**	**1.04 (0.04, 2.04)** ^f^
UVM	0.1	0.4	0.44	—	—	—	—	−**6.15**	−0.1	**7.08**	1.45	**<0.001**	−0.01 (−0.40, 0.37)^e^

*ACC* adrenocortical carcinoma, *BLCA* bladder urothelial carcinoma, *BRCA* breast invasive carcinoma, *CESC* cervical squamous cell carcinoma and endocervical adenocarcinoma, *CHOL* cholangiocarcinoma, *COAD* colon adenocarcinoma, *DLBC* lymphoid neoplasm diffuse large B-cell lymphoma, *ESCA* oesophageal carcinoma, *GBM* glioblastoma multiforme, *HNSC* head and neck squamous cell carcinoma, *KICH* kidney chromophobe, *KIRC* kidney renal clear cell carcinoma, *KIRP* kidney renal papillary cell carcinoma, *LAML* acute myeloid leukaemia, *LGG* brain lower grade glioma, *LIHC* liver hepatocellular carcinoma, *LUAD* lung adenocarcinoma, *LUSC* lung squamous cell carcinoma, *MESO* mesothelioma, *OV* ovarian serous cystadenocarcinoma, *PAAD* pancreatic adenocarcinoma, *PCPG* pheochromocytoma and paraganglioma, *PRAD* prostate adenocarcinoma, *READ* rectum adenocarcinoma, *SARC* sarcoma, *SKCM* skin cutaneous melanoma, *STAD* stomach adenocarcinoma, *TGCT* testicular germ cell tumours, *THCA* thyroid carcinoma, *THYM* thymoma, *UCEC* uterine corpus endometrial carcinoma, *UCS* uterine carcinosarcoma, *UVM* uveal melanoma.

^a^*P*-values < 0.05, its corresponding effect size and 95% confidence intervals, and adjusted residuals that exceed |± 2| are bolded.

^b^*P*-value was calculated using Student’s *t*-test.

^c^*P-*value of comparisons between TCGA and SEER (sex, stage at diagnosis) or NAACCR (race) cases was using the Chi-square test. For CHOL, KICH, UCS, and UVM, Fisher’s exact test was used for the comparison of stage at diagnosis.

^d^Adjusted residuals.

^e^Generalised linear models adjusted for age at diagnosis, sex, race, and stage at diagnosis.

^f^Generalised linear models adjusted for age at diagnosis, race, and stage at diagnosis.

^g^Generalised linear models adjusted for age at diagnosis, sex, and race.

^h^Generalised linear models adjusted for age at diagnosis and race

### Sex

For most cancer types (22/27), the observed sex distribution for TCGA cases was similar to SEER cases. Lung squamous cell carcinoma (LUSC), skin cutaneous melanoma (SKCM), and thyroid carcinoma (THCA) had a significantly higher proportion of male cases (74.0% vs. 62.4%, 61.7% vs. 56.6%, and 26.8% vs. 22.8%, respectively), while LIHC and SARC cases had an excess of female cases (32.4% vs. 22.6%, 54.4% vs. 46.7%) in TCGA vs. SEER (Tables 1 and 3).

### Race

**Fig. 2:**
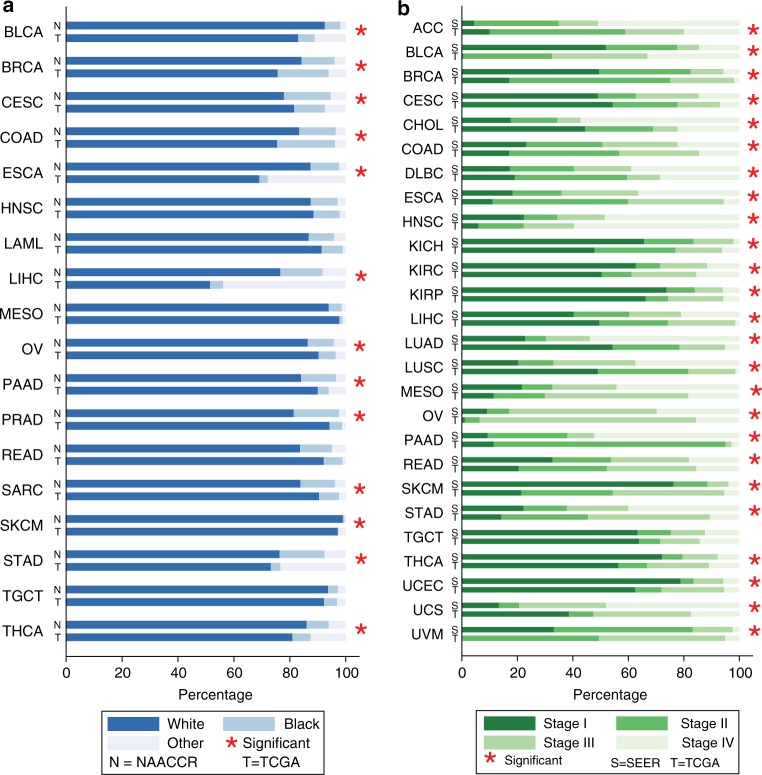
Race and stage proportion difference. **a** Race proportion of TCGA and NAACCR cases. **b** Stage proportion of TCGA and SEER cases. The stars indicate the difference was statistically significant at an alpha threshold *P* < 0.05

Overall, compared to the NAACCR cases, individuals whose reported race was Other (Asian, American Indian, or Alaska Native) were over-represented in TCGA. The observed race distribution was disproportional for 13/18 cancer types (Fig. 2a). Among the 13 cancers, eight (bladder urothelial carcinoma (BLCA), BRCA, ESCA, LIHC, pancreatic adenocarcinoma (PAAD), SKCM, STAD, and THCA) had a significantly higher percentage (adjusted residuals ≥ 2) of individuals with reported Other race (Asian, American Indian, or Alaska Native) and eight (cervical squamous cell carcinoma and endocervical adenocarcinoma (CESC), ESCA, LIHC, OV, PAAD, PRAD, SARC, and STAD) had a lower percentage (adjusted residuals ≤ −2) of reported Black race in TCGA vs. NAACCR (Table 3).

### Stage at diagnosis

For the 26 TCGA cancer types with stage information, evidence for stage dissimilarities was observed for most cancer types (25/26) (Fig. 2b). Specifically, compared to SEER cases, 16 cancers had a significantly lower proportion of stage I in the TCGA cohort, 19 cancers had a significantly higher proportion of stage II, 12 cancers had a significantly higher proportion of stage III, and 14 cancers had a significantly lower proportion of stage IV (Table 3).

### Survival months

**Fig. 3:**
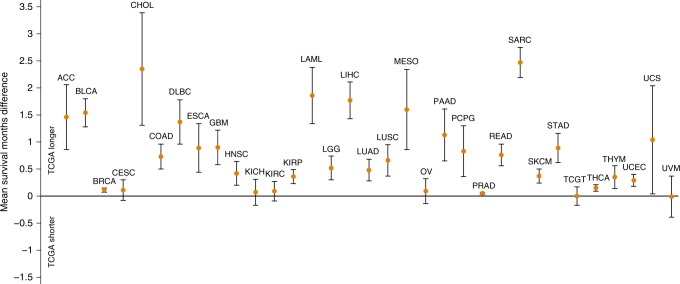
Survival months difference between TCGA and SEER cases. Mean survival months difference at 12-months of follow-up with corresponding 95% CIs. Dots indicate the mean survival months difference and lines represent its 95% CIs. The *y*-axis below zero indicates TCGA cases with a shorter survival time than SEER cases

Using 12 months as an end point, the adjusted mean all-cause survival months were significantly longer for cases with 27/33 cancer types in TCGA relative to SEER. For the remaining six cancer types (CESC, KICH, KIRC, OV, testicular germ cell tumours (TGCT), and UVM), no statistically significant difference was found (Fig. 3). It is noteworthy that for CHOL and SARC, TCGA cases lived an average of over 2 months (2.35 and 2.47 months, respectively) longer than SEER cases after 12 months of follow-up (Table 3).

## Discussion

In this study, we observed that despite an approximately equal sex distribution for most cancer types included in TCGA vs. SEER data, differences exist in mean diagnosis age, race, stage at diagnosis distributions, and mean survival months. Generally, our analysis indicates that TCGA cases are younger and survive longer than those from SEER.

A previous study comparing the characteristics of TCGA cases to the U.S. general population was conducted by Spratt et al.^15^ The authors reported that TCGA cases with 10 cancer types compared to the U.S. population were 77% vs. 64% White, 12% vs. 12% Black, 3% vs. 5% Asian, 3% vs. 16% Hispanic, and 0.5% vs. 1–2% Native Hawaiian, Pacific Islander, Alaskan Native, or American Indian descent. White cases were over-represented and Asian and Hispanic cases were under-represented compared to the general population. However, the Spratt et al. study used the general U.S. population as the comparator, which is different from the composition of U.S. cancer patients who are one of the prime beneficiaries of TCGA results.

Another more recent study compared the distribution of TCGA cases  by age to SEER cases  for  nine cancer types.^16^ Similar to our study, the age distributions for cases in the SEER database were skewed older than those in the TCGA data for nearly all cancer types examined. Specifically, TCGA cases  < 70 years were well represented across most tumour types, but  cases aged 80–99 years were under-represented for all cancers. These data are also consistent with that from clinical trials.^30^ TCGA specimens are primarily from U.S. academic institutions,^3,15^ suggesting that younger patients are more likely to be seen at academic centres and participate in research where the samples were acquired. A systematic review on the recruitment of older cancer patients to clinical trials reported that age is a significant barrier to recruitment.^31^ For example, Kemeny et al. found that 68% of younger stage II breast cancer patients were offered a trial vs. 34% of the older patients (*P* < 0.001).^32^ It is presumed that older patients may need extra time and resources to access available clinical trials or they are often excluded because they do not meet eligibility criteria.^31^ Our results emphasize the importance of increasing access of older cancer patients to cancer genomic projects to increase the applicability of the findings to these patients.

Racial disparities in cancer incidence and survival have been well documented among various cancers. Although socioeconomic and cultural differences that differ between racial groups can explain some of the disparities, recent progress in cancer genomic sequencing allows for a molecular understanding.^33,34^ Genomic landscape differences that co-vary by race, a marker of ancestry, may influence cancer treatment. For example, one study reported that even after adjusting for smoking status and sex, race was still significantly associated with *EGFR* mutations.^35^ *EGFR* mutations were highly prevalent in Asians at 30% vs. 7% in Whites.^36^ In addition, results from a meta-analysis of randomised controlled trials have reported that compared with Caucasians, Asians have a higher survival and response rate to chemotherapy.^37^ In our study, the race distribution was notably dissimilar for 13/18 cancers, with 8/13 cancers having under-representation in individuals with Black race, which may translate to a distinct genomic landscape that may be under-represented for many cancer types. Notably, 8 of these 13 cancers had higher representation by individuals with Other race (Asian, American Indian, or Alaska Native). This over-representation may be due to TCGA cancers with small sample sizes where a relatively large proportion can be found even only with few cases in the Other population.

Stage is a well-established predictor of cancer prognosis and survival.^38^ Studies have also reported notable genetic variation in cancers by stage.^39,40^ In our study, stage dissimilarities existed for almost all cancer types (25/26). However, these identified differences between datasets may be due to the fact that only individuals  who had a resection procedure were included in TCGA.^14^ Individuals with unresectable cancers, such as cancers with advanced stage or metastatic cancer,^41^ did not meet the inclusion criteria of the program, which likely led to a lower stage distribution of the cases in TCGA compared to SEER. In addition, other differences may have contributed to stage differences including the sample eligibility requirements of only untreated first primary tumour samples being fresh frozen.^14^

To our knowledge, this is the largest study to compare clinical characteristics of TCGA cancer cases to a sample of the general population of U.S. cancer  cases. However, our study has limitations. No specific diagnosis criteria for each cancer type have been published for TCGA to our knowledge. Thus, the corresponding cancers in SEER were matched by cancer site and histology, and identified by ICD-O-3 primary site and histology/behavior code. Moreover, cases with certain cancers had missing race, stage, and survival months information. Particularly, 6/33 TCGA cancer types (THCA, LUSC, PRAD, COAD, READ, and UVM) had over 15% missing data on race, and 5/26 SEER cancers (BLCA, LIHC, MESO, CHOL, and UVM) had over 15% missing data on stage. In addition, for the race comparison, only 18 cancers in NAACCR were identified with sites matching to those of TCGA cases.

In conclusion, we found dissimilarities in the distributions of demographic and clinical characteristics between TCGA and general population cancer cases for the majority of cancers. Increased awareness of under-represented groups by researchers conducting cancer genomic research will allow for targeted efforts that increase the representativeness of genomic data that is important for precision medicine.

## Electronic supplementary material

Supplementary Table 1: Cancer Types in TCGA, SEER and NAACCR

## Availability of data and materials

TCGA: https://portal.gdc.cancer.gov/ SEER: www.seer.cancer.gov NAACCR: https://faststats.naaccr.org/

## References

[1] Lawrence MS (2014). Discovery and saturation analysis of cancer genes
across 21 tumour types. Nature.

[2] Saha SK (2015). Corrigendum: mutant IDH inhibits HNF-4alpha to block
hepatocyte differentiation and promote biliary cancer. Nature.

[3] Tomczak K, Czerwinska P, Wiznerowicz M (2015). The Cancer Genome Atlas (TCGA): an immeasurable source
of knowledge. Contemp. Oncol..

[4] National Institute of Health, National Cancer Institute, National Human Genome Research Institute. TCGA program overview. http://cancergenome.nih.gov/abouttcga/overview (2016).

[5] Calvo E, Baselga J (2006). Ethnic differences in response to epidermal growth
factor receptor tyrosine kinase inhibitors. J. Clin. Oncol..

[6] Shi Y (2014). A prospective, molecular epidemiology study of EGFR
mutations in Asian patients with advanced non-small-cell lung cancer of
adenocarcinoma histology (PIONEER). J. Thorac. Oncol..

[7] Kurian AW (2010). BRCA1 and BRCA2 mutations across race and ethnicity:
distribution and clinical implications. Curr. Opin. Obstet. Gynecol..

[8] Cote ML (2012). Racial differences in oncogene mutations detected in
early-stage low-grade endometrial cancers. Int. J. Gynecol. Cancer.

[9] Keenan T (2015). Comparison of the genomic landscape between primary
breast cancer in African American versus white women and the association of
racial differences with tumour recurrence. J. Clin. Oncol..

[10] Tan DS, Mok TS, Rebbeck TR (2016). Cancer genomics: diversity and disparity across
ethnicity and geography. J. Clin. Oncol..

[11] Dresler CM (2000). Gender differences in genetic susceptibility for lung
cancer. Lung Cancer.

[12] Hwang SJ, Lozano G, Amos CI, Strong LC (2003). Germline p53 mutations in a cohort with childhood
sarcoma: sex differences in cancer risk. Am. J. Hum. Genet..

[13] Liu L, Zhang J, Wu AH, Pike MC, Deapen D (2012). Invasive breast cancer incidence trends by detailed
race/ethnicity and age. Int. J. Cancer.

[14] National Institute of Health, National Cancer Institute, National Human Genome Research Institute. TCGA tissue sample requirements: high quality requirements yield high quality data. https://cancergenome.nih.gov/cancersselected/biospeccriteria (2018).

[15] Spratt DE (2016). Racial/ethnic disparities in genomic
sequencing. JAMA Oncol..

[16] Wahl DR (2017). Pan-cancer analysis of genomic sequencing among the
elderly. Int. J. Radiat. Oncol. Biol. Phys..

[17] Surveillance, Epidemiology, and End Results (SEER) Program (www.seer.cancer.gov) SEER*Stat Database: Incidence - SEER 9 Regs Research Data, Nov 2017 Sub (1973-2015) <Katrina/Rita Population Adjustment> - Linked To County Attributes - Total U.S., 1969-2016 Counties, National Cancer Institute, DCCPS, Surveillance Research Program, released April 2018, based on the November 2017 submission.

[18] Weir HK (2014). Evaluation of North American Association of Central
Cancer Registries’ (NAACCR) data for use in population-based cancer survival
studies. J. Natl Cancer Inst. Monogr..

[19] National Institute of Health, National Cancer Institute. NCI genomic data commons data portal. https://portal.gdc.cancer.gov/ (2016).

[20] North American Association of Central Cancer Registries. NAACCR fast stats: an interactive tool for quick access to key NAACCR cancer statistics. http://www.naaccr.org/ (2016).

[21] Surveillance Epidemiology and End Results (SEER) Program. About the SEER registries. https://seer.cancer.gov/registries/ (2016).

[22] North American Association of Central Cancer Registries. Cancer in North America CiNA volumes. https://www.naaccr.org/cancer-in-north-america-cina-volumes/ (2016).

[23] Surveillance Epidemiology and End Results (SEER) Program. Survival time calculation. https://seer.cancer.gov/survivaltime/SurvivalTimeCalculation.pdf (2016).

[24] Fritz CO, Morris PE, Richler JJ (2012). Effect size estimates: current use, calculations, and
interpretation. J. Exp. Psychol. Gen..

[25] Royston P, Parmar MK (2013). Restricted mean survival time: an alternative to the
hazard ratio for the design and analysis of randomized trials with a
time-to-event outcome. BMC Med. Res. Methodol..

[26] Zhao L (2016). On the restricted mean survival time curve in survival
analysis. Biometrics.

[27] A’Hern RP (2016). Restricted mean survival time: an obligatory end point
for time-to-event analysis in cancer trials?. J. Clin. Oncol..

[28] Andersen PK, Perme MP (2010). Pseudo-observations in survival
analysis. Stat. Methods Med. Res..

[29] Andersen PK, Hansen MG, Klein JP (2004). Regression analysis of restricted mean survival time
based on pseudo-observations. Lifetime Data Anal..

[30] Murthy VH, Krumholz HM, Gross CP (2004). Participation in cancer clinical trials: race-, sex-,
and age-based disparities. J. Am. Med. Assoc..

[31] Townsley CA, Selby R, Siu LL (2005). Systematic review of barriers to the recruitment of
older patients with cancer onto clinical trials. J. Clin. Oncol..

[32] Kemeny MM (2003). Barriers to clinical trial participation by older
women with breast cancer. J. Clin. Oncol..

[33] Burchard EG (2003). The importance of race and ethnic background in
biomedical research and clinical practice. N. Engl. J. Med..

[34] El-Telbany A, Ma PC (2012). Cancer genes in lung cancer: racial disparities: are
there any?. Genes Cancer.

[35] Bauml J (2013). Frequency of EGFR and KRAS mutations in patients with
non small cell lung cancer by racial background: do disparities
exist?. Lung Cancer.

[36] Zhou W, Christiani DC (2011). East meets West: ethnic differences in epidemiology
and clinical behaviors of lung cancer between East Asians and
Caucasians. Chin. J. Cancer.

[37] Soo RA (2011). Ethnic differences in survival outcome in patients
with advanced stage non-small cell lung cancer: results of a meta-analysis of
randomized controlled trials. J. Thorac. Oncol..

[38] Naruke T, Goya T, Tsuchiya R, Suemasu K (1988). Prognosis and survival in resected lung carcinoma
based on the new international staging system. J. Thorac. Cardiovasc. Surg..

[39] Blaveri E (2005). Bladder cancer stage and outcome by array-based
comparative genomic hybridization. Clin. Cancer Res..

[40] Richter J (1997). Marked genetic differences between stage pTa and stage
pT1 papillary bladder cancer detected by comparative genomic
hybridization. Cancer Res..

[41] Balaban EP (2016). Locally advanced, unresectable pancreatic cancer:
American society of clinical oncology clinical practice guideline. J. Clin. Oncol..

