# Supplementary material for: Characteristics of The Cancer Genome Atlas cases relative to U.S. general population cancer cases
Source: Br J Cancer. 2018 Aug 21;119(7):885–92. doi: 10.1038/s41416-018-0140-8 (PMC6189215; doi:10.1038/s41416-018-0140-8)
Supplement: Supplementary file 1 — Supplementary Table 1: Cancer Types in TCGA, SEER and NAACCR [file 41416_2018_140_MOESM1_ESM.docx]

| **Supplementary Table 1**: Cancer types in TCGA, SEER and NAACCR | | |
| --- | --- | --- |
| **TCGA cancer types** | **SEER ICD-O-3**  **Site (histology) code** | **NAACCR Site** |
| Adrenocortical carcinoma (ACC) | C74.0-C74.9 (8370/3) | NA |
| Bladder urothelial carcinoma (BLCA) | C67.0-C67.9 (8050, 8120, 8130, 8131)^1^ | Urinary bladder (invasive & in situ) |
| Breast invasive carcinoma (BRCA) | C50.0-C50.9 (801-857)^2,3^ | breast |
| Cervical squamous cell carcinoma and endocervical adenocarcinoma (CESC) | C53.0-C53.9 (807, 814-857) | Cervix uteri |
| Cholangiocarcinoma (CHOL) | C22.1 (8160/3) | NA |
| Colon adenocarcinoma (COAD) | C18.0-C18.9 (814-857) | colon |
| Lymphoid neoplasm diffuse large B-cell lymphoma (DLBC) | (9678-9684)^4^* | NA |
| Oesophageal carcinoma (ESCA) | C15.0-C15.9 (801-857) | Oesophagus |
| Glioblastoma multiforme (GBM) | (9440/3)^5^* | NA |
| Head and neck squamous cell carcinoma (HNSC) | C00.0-C15.0, C15.3, C30.0, C31.0-C33.9, C41.0, C41.1, C47.0, C49.0, C73.9, C75.4 C77.0 (807)^6^ | Larynx, oral cavity and pharynx, lip, tongue, salivary gland, floor of mouth, gum & other mouth, oropharynx & tonsil |
| kidney chromophobe (KICH) | C64.9, C65.9 (8317, 8270)^7^ | NA |
| Kidney renal clear cell carcinoma (KIRC) | C64.9, C65.9 (8310/3)^7^ | NA |
| Kidney renal papillary cell carcinoma (KIRP) | C64.9, C65.9 (8260)^7^ | NA |
| Acute myeloid leukemia (LAML) | ICCC site recode ICD-O-3/WHO 2008=I(b) Acute myeloid leukemias | Acute myeloid leukemia |
| Brain lower grade glioma (LGG) | C71.0-C71.9 (9380-9384, 9391-9460, 9480, Grade I and II)^8,9^ | NA |
| Liver hepatocellular carcinoma (LIHC) | C22.0 (817-818) | Liver and intrahepatic bile duct |
| Lung adenocarcinoma (LUAD) | C34.0-C34.9 (814-857) | NA |
| Lung squamous cell carcinoma (LUSC) | C34.0-C34.9 (807) | NA |
| Mesothelioma (MESO) | (905)* | Mesothelioma |
| Ovarian serous cystadenocarcinoma (OV) | C56.9 (8441.3) | Ovary |
| Pancreatic adenocarcinoma (PAAD) | C25.0-C25.9 (814-857) | Pancreas |
| Pheochromocytoma and paraganglioma (PCPG) | (868, 869, 870)* | NA |
| Prostate adenocarcinoma (PRAD) | C61.9 (814-857) | Prostate |
| Rectum adenocarcinoma (READ) | C20.9 (814-857) | Rectum |
| Sarcoma (SARC) | (880)* | Soft tissue including heart |
| Skin cutaneous melanoma (SKCM) | C44.0-C44.9 (8720-8790) | Melanoma of the skin |
| Stomach adenocarcinoma (STAD) | C16.0-C16.9 (814-857) | Stomach |
| Testicular germ cell tumors (TGCT) | C62.0-C62.9 (9060-9100) | Testis |
| Thyroid carcinoma (THCA) | C73.9 (801-857) | Thyroid |
| Thymoma (THYM) | C37.9 (8580-8585)^10^ | NA |
| Uterine corpus endometrial carcinoma (UCEC) | C54.0-C54.9 (8140, 8380, 8382, 8480, 8482, 8560, 8570, 8310, 8441, 8460, 8260)^11^ | NA |
| Uterine carcinosarcoma (UCS) | C55.9 (8980-8981) | NA |
| Uveal melanoma (UVM) | C69.2-C69.4 (8720/3)^12^ | NA |

NA: not applicable

*No site code needed.

**References**

1. Wright JL, Black PC, Brown GA, Porter MP, Kamat AM, Dinney CP, et al. Differences in survival among patients with sarcomatoid carcinoma, carcinosarcoma and urothelial carcinoma of the bladder. J Urol 2007;178(6):2302-7.

2. Anderson WF, Devesa SS. In situ male breast carcinoma in the Surveillance, Epidemiology, and End Results database of the National Cancer Institute. Cancer 2005;104(8):1733-41.

3. Rv H. Breast Cancer. Cancer 1995;75:257-69.

4. Shenoy PJ, Malik N, Nooka A, Sinha R, Ward KC, Brawley OW, et al. Racial differences in the presentation and outcomes of diffuse large B‐cell lymphoma in the United States. Cancer 2011;117(11):2530-40.

5. Bauchet L, Mathieu-Daudé H, Fabbro-Peray P, Rigau V, Fabbro M, Chinot O, et al. Oncological patterns of care and outcome for 952 patients with newly diagnosed glioblastoma in 2004. Neuro Onco 2010; 12(7): 725-35.

6. Davies L, Welch HG. Epidemiology of head and neck cancer in the United States. Otolaryngol Head Neck Surg. 2006;135(3):451-7. e3.

7. Olshan AF, Kuo TM, Meyer AM, Nielsen ME, Purdue MP, Rathmell WK. Racial difference in histologic subtype of renal cell carcinoma. Can Med 2013;2(5):744-9.

8. Grier JT, Batchelor T. Low-grade gliomas in adults. The Oncologist 2006;11(6):681-93.

9. Ostrom QT, Gittleman H, Farah P, Ondracek A, Chen Y, Wolinsky Y, et al. CBTRUS statistical report: Primary brain and central nervous system tumors diagnosed in the United States in 2006-2010. Neuro Onco 2013;15(suppl 2):ii1-ii56.

10. Weksler B, Shende M, Nason KS, Gallagher A, Ferson PF, Pennathur A. The role of adjuvant radiation therapy for resected stage III thymoma: a population-based study. Ann Thorac Surg. 2012;93(6):1822-9.

11. Felix AS, Weissfeld JL, Stone RA, Bowser R, Chivukula M, Edwards RP, et al. Factors associated with Type I and Type II endometrial cancer. Cancer Causes Control 2010;21(11):1851-6.

12. Singh AD, Turell ME, Topham AK. Uveal melanoma: trends in incidence, treatment, and survival. Ophthalmology 2011;118(9):1881-5.
